# Outcome of endoscopic vacuum therapy for duodenal perforation

**DOI:** 10.1007/s00464-022-09686-w

**Published:** 2022-10-14

**Authors:** Mickael Chevallay, Florian Lorenz, Philippe Bichard, Jean-Louis Frossard, Thomas Schmidt, Tobias Goeser, Christiane Josephine Bruns, Stefan P. Mönig, Seung-Hun Chon

**Affiliations:** 1 Department of Visceral Surgery, Geneva University Hospitals, Geneva, Switzerland; 2 Department of Gastroenterology and Hepatology, University Hospital of Cologne, Cologne, Germany; 3 Department of Gastroenterology and Hepatology, Geneva University Hospital, Geneva, Switzerland; 4 Department of General, Visceral, Cancer, and Transplantat Surgery, University Hospital of Cologne, Cologne, Germany; 5 Department of Gastroenterology and Hepatology, Department of General, Visceral, Cancer, and Transplantat Surgery, Interdisciplinary Endoscopy Unit of University Hospital Cologne, Kerpener Street 62, 50937 Cologne, Germany

**Keywords:** Duodenal perforation, Endoscopic vacuum therapy, Leak management, Upper gastrointestinal surgery, Complication management

## Abstract

**Background:**

Duodenal defects are complex clinical situations, and their management is challenging and associated with high mortality. Besides surgery, endoscopic treatment options exist, but the size and location of the perforation can limit their application. We present a retrospective study, demonstrating a successful application of endoscopic vacuum therapy (EVT) for duodenal leaks.

**Methods:**

We performed a retrospective study of all patients who underwent EVT for duodenal perforations between 2016 and 2021 at two tertiary centers. We analyzed demographic and clinical patient characteristics, surgical outcomes, leak characteristics, sponge-related complications, and success rate.

**Results:**

Indications for treatment with EVT in the duodenum consisted of leak after duodenal suture of a perforated ulcer (*n* = 4), iatrogenic perforation after endoscopic resection (*n* = 2), iatrogenic perforation during surgery (*n* = 2), and anastomotic leak after upper gastrointestinal surgery (*n* = 2). EVT was used as a first-line treatment in seven patients and as a second-line treatment in three patients. EVT was successfully applied in all interventions (*n* = 10, 100%). Overall, EVT lead to definitive closure of the defects in eight out of ten patients (80%). No severe EVT-related adverse events occurred.

**Conclusion:**

EVT is safe and technically feasible, so it emerges as a promising endoscopic treatment option for duodenal leaks. However, multidisciplinary collaboration and management are important to reduce the occurrence of postoperative complications, and to improve recovery rates.

Duodenal defects are complex clinical situations, and their management is challenging. The reported prevalence of leaks after duodenal ulcer suturing ranges between 1 and 13% [1–3]. Duodenal perforations after endoscopic retrograde cholangiopancreatography are rare, occurring in 0.08–1.6% of cases [4, 5]. However, the prevalence of this complication is much higher after endoscopic mucosal resection, ranging from 6 to 13% [6, 7]. Overall mortality associated with iatrogenic perforation can reach 20% [8]. Various therapeutic strategies have been developed: from surgical repair to a variety of endoscopic interventional techniques [9–13]. The use of clips is an established therapeutic option, but recently, endoscopic vacuum therapy (EVT) has become a promising alternative [14].

Treatment time with EVT can be short, and the application of suction force directly accelerates the granulation process [15, 16]. There are two different methods for EVT application: intraluminal EVT involves placing a sponge in the gastrointestinal tract, while intracavitary EVT involves placing the sponge in a paraintestinal wound cavity. During intraluminal EVT, the esophageal passage is completely blocked, preventing food and drink intake and thus requiring a nasojejunal tube to ensure enteral feeding [17].

Wedemeyer et al. described the first use of EVT for upper intestinal leaks in 2008 with successful treatment of two patients with intrathoracic anastomotic leaks after esophagectomy [18]. Since then, EVT has shown its effectiveness on a larger scale for esophageal leaks and complications after bariatric procedures, achieving promising success rates [14].

Reports of the use of EVT for duodenal defects are less common. The distance from a natural orifice poses a technical challenge for the placement. Application of EVT for duodenal perforations has so far been described in only one case series and three case reports [19–21].

Therefore, our goal was to evaluate the safety, rate of success and morbidity of EVT in the management of duodenal perforations at two tertiary centers.

## Methods

### Study design

This retrospective study was performed at the Interdisciplinary Endoscopy Unit of the Department of Gastroenterology and Hepatology, the Department of General, Visceral, Cancer, and Transplantation Surgery at the University Hospital Cologne, and at the Department of Visceral Surgery and Gastroenterology at the Geneva University Hospitals. Data were retrieved from our prospectively maintained endoscopic surgery database “Clinic WinData” (version 8.06; E&L medical system GmbH, Erlangen, Germany) and from our hospital database “Orbis” (version 08043101; Agfa HealthCare N.V., Belgium), as well as from the Geneva University Hospitals. The following information was collected: demographic and clinical patient characteristics, details of the disease, surgical outcome data, endoscopic findings, and sponge-related complications.

In our study, we included all patients with an iatrogenic or post-interventional duodenal leak diagnosed through an endoscopic examination between January 2016 and December 2021. All patients underwent EVT with the EsoSponge® (Aeskulap AG, Melsung, Germany) at our hospitals.

### Ethics declarations

The manuscript was submitted to the ethics committee at the University Hospital Cologne, which exempted us from applying for ethical approval as, under German law, no separate application and no ethical approval are required for purely retrospective studies.

*We also submitted the manuscript to the ethics committee at the Geneva University Hospitals. This study was considered as falling outside of the scope of the Swiss legislation regulating research on human subjects, so that the need for local ethics committee approval was waived.*

### Leak detection and management

In the event of a clinically suspected duodenal leak, a flexible video esophagogastroduodenoscopy (e.g., Pentax Medical, Japan; Olympus Corporation, Tokyo, Japan) was performed. If a leak could not be diagnosed with certainty, additional diagnostic tools were used (e.g., computed tomography scan and contrast swallow). The examination was performed under general anesthesia (for intubated intensive care unit patients); a combination of midazolam (e.g., Roche Pharma AG, Germany) and propofol (e.g., Fresenius Kabi Germany GmbH); or only propofol.

### Endoscopic vacuum therapy

Open-pore polyurethane foam (EsoSponge, Aesculap AG, Germany) was adapted individually to the size of the leak. Using an endoscope to ensure constant observation, the polyurethane sponge mounted on a drainage tube was moved to the desired location. A pyloric dilatation was performed to facilitate the passage of the sponge. Once the correct position was verified, the drainage tube was moved from the oral to the nasal cavity. The endoscopist then connected the drainage tube to an electric vacuum pump (e.g., VivanoTec®, Hartmann AG, Germany) and applied a continuous negative pressure of 125 mmHg.

No additional diagnostic checks were routinely performed after the insertion of the sponge. However, if the vacuum pump malfunctioned, an endoscopy was performed immediately to check whether sponge replacement was necessary. The size of the leak and the wound cavity behind the leak determined the treatment duration. *Therapy was only considered complete when the leak was closed, or the wound cavity lined with granulation tissue as this was a sign of local healing associated with clinical leak closure.* The interval between the EVT sponge changes was 3–5 days. Oral intake was not possible during EVT. During EVT sponge changes, the leak site was endoscopically evaluated for persisting or healed defect. In the case of successful healing, the therapy was terminated, and the patient was put on an oral diet, starting with drinking water on the same day. Patients stayed in the hospital for the duration of the EVT treatment.

### Additional treatment

The endoscopist performed additional non-operative management. A double-lumen nasogastric feeding tube (e.g., Freka® Sonde, Fresenius Kabi Germany GmbH) or a triple-lumen diverted nasogastric feeding tube (e.g., Freka® Trelumina, Fresenius Kabi Germany GmbH) was endoscopically placed directly before or after the intervention. The gastric lumen was used to decompress the duodenal region and to evacuate the gastric reflux. Enteral caloric nutrition was provided via the duodenal lumen. The nasojejunal tube remained, if tolerated, until the leak was sealed successfully. Further treatment involved intravenous administration of antimicrobials (including antifungals). In the event of mediastinal, pleural or abdominal fluid collection, an external drainage was applied interventionally, either ultrasonically guided or computed tomography-guided.

### Statistics

Distributions of quantitative variables are described as median and interquartile range (IQR). They were compared using the Mann–Whitney *U* test. Qualitative variables are summarized by count and percentage. We compared the groups using the Fisher’s exact test.

A two-tailed *p* < 0.05 was considered statistically significant. Due to the small number of cases, multivariate analysis was not performed. Data were analyzed in SPSS Statistics version 25 (IBM Corp., Armonk, NY, USA) for Windows (Microsoft Corp, Redmond, WA, USA) and Microsoft Excel Version 2013 for Windows (Microsoft Corp, Redmond, WA, USA).

## Results

Between 2016 and 2021, ten patients with duodenal defects were treated with EVT, five in each of the two centers. Complete leak closure was achieved in eight patients (80%). Details of the patients’ baseline characteristics and procedural data are shown in Table 1.

**Table 1:** Patients’ characteristics and EVT outcomes

	All patients (*n* = 10)
Age, median (IQR)	65.5 (60.5–74)
Female, n (%)	1 (10%)
BMI, median (IQR)	24 (IQR: 22.75–29)
ASA scores, n (%)	1: 0
	2: 2 (20%)
	3: 4 (40%)
	4: 4 (40%)
Etiology of the perforation	Leak after duodenal suture: 4 (40%)
	Anastomotic leak 2 (20%)
	Iatrogenic perforation 2 (20%)
	After EMR 2 (20%)
Prior treatment	Yes 3 (30%)
	No 7 (70%)
Latency between leak identification and EVT initiation, days, median (IQR)	1 (0–8)
Length of stay, days, median (IQR)	78 (22–86)
Size of the defect, centimeters, median (IQR)	3 (2–3.5)
Localization of the EVT, median (IQR)	Intraluminal 8 (80%)
	Intracavitary 2(20%)
EVT duration, days, median (IQR)	9 (7–30.75)
Number of EVT changes, median (IQR)	3 (2–5)
Additional treatment of leak (surgical or radiological drainage), *n* (%)	Yes 9 (90%)
	No 1 (10%)
Length of ICU stay, median (IQR)	21 (10.5–36.75)
In hospital complications (Clavien Dindo > 3a), *n* (%)	3 (30%)
Success of EVT, *n* (%)	8 (80%)
Mortality, *n* (%)	2 (20%)

*EMR* Endoscopic mucosal resection, *BMI* Body Mass Index, *ASA* American Society of Anesthesiologists, *ICU* Intensive Care Unit

The median age of the patients was 65.5 years (IQR: 60.5–74) and only one patient was female. The etiology of the duodenal defects were: a leak after suture for ulcer perforation in four patients, and after endoscopic resection in two patients; an anastomotic leak in two patients; and an iatrogenic lesion in two patients (one after cholecystectomy and one after intraperitoneal onlay mesh). Two patients had an oncological condition as a primary indication for surgery (one duodenal neuroendocrine tumor and one duodenal adenocarcinoma). The mean body mass index (BMI) was 24 kg/m^2^ (IQR: 22.75–29). Two patients regularly consumed nicotine. One patient had a COVID-19 infection on admission. The ASA score was 2 in two patients, 3 in four patients, and 4 in four patients.

The leak was detected at a median 6.5 days after the surgery or the endoscopic treatment (IQR: 5.5–10.25). The median diameter of the duodenal defect estimated by endoscopy was 2.5 cm (IQR: 2–5). Additional radiologic drainage of the leak was performed in five patients. Seven patients needed to be monitored in the intensive care unit (ICU) over a median stay of 21 days (IQR: 10.5–31.5).

**Fig. 1:**
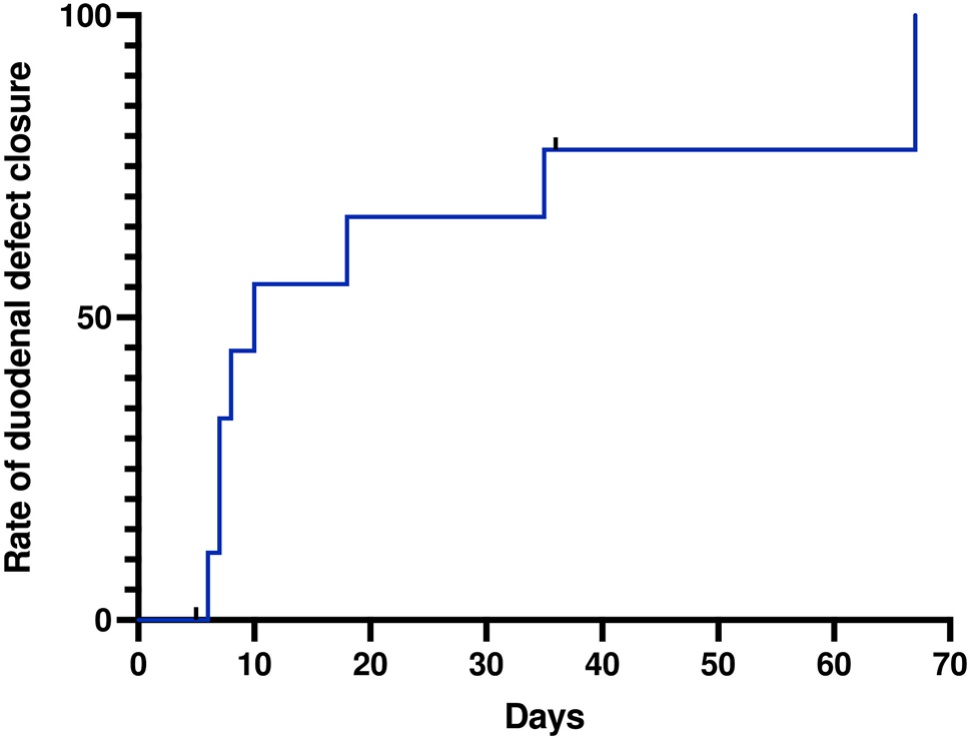
Kaplan–Meier analysis of duodenal defect closure over time

**Fig. 2:**
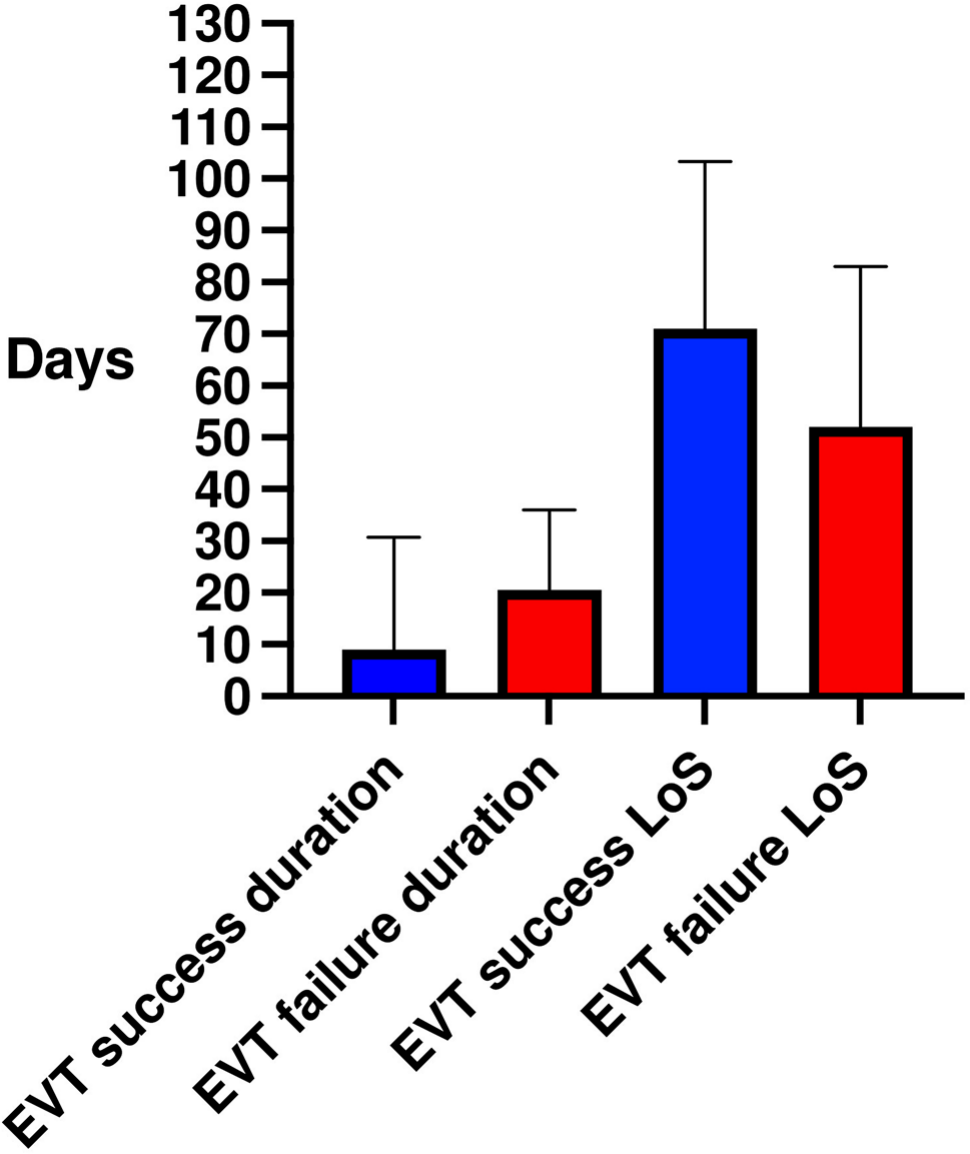
Median and interquartile range of EVT duration and length of stay (LoS) in patients with successful and failed EVT

The median duration of the EVT was 9 days (IQR: 7–30.75). Figure 1 shows the Kaplan–Meier estimate of closure rate for the duodenal defects. The median length of hospital stay was 68.5 days (IQR: 26.5–78) (Fig. 2). Prior to the EVT, closure was attempted in five patients: with surgery (direct suture of the perforation) in four patients, and endoscopically, with an over-the-scope clip, in one. In the four patients who underwent attempted surgical repair, the delay from admission to surgical management was a median of 1 day (IQR: 0.75–2).

Complications during EVT occurred in two of the ten patients: a right-sided pneumothorax in one case and bleeding in the other. The pneumothorax was successfully treated with surgical drainage. The bleeding resolved itself after the removal of the sponge. Complications after EVT were observed in three patients who had intraabdominal collections that needed radiologic drainage.

EVT failed in two patients: one from each center. One failure was observed in a 62-year-old male with liver cirrhosis and obesity. He suffered a leak 5 days after the suture of a duodenal ulcer. Endoscopy confirmed a duodenal defect of 3.5 cm. EVT was started a day after the discovery of the leak, but it failed to completely close the defect, with a persistent fistula output after 11 EVT changes. EVT was then stopped, and the patient underwent surgical drainage of retroperitoneal collection, which finally closed the defect.

The other patient was a 64-year-old male who suffered a duodenal ulcer perforation of one centimeter. He was classified as ASA 4 and was too fragile to undergo surgical repair. EVT was attempted in parallel with radiologic drainage. One sponge change was performed. After the explanation of the second EVT, no healing was noted. After a thorough discussion with the patient and his family, he decided to withdraw from care. The EVT was stopped while the patient suffered multiple organ failure due to sepsis. The patient deceased after 21 days of therapy.

This patient was one of two in our study who deceased. In the second patient, the evolution of the duodenal defect was favorable, showing a complete closure during the last endoscopy. However, the patient died from uncontrolled sepsis after 36 days of hospitalization.

When comparing the two groups (EVT success and EVT failure), there were no statistical differences in age, length of stay, etiology of the perforation, extent of the leak and number of EVT changes (Table 2).

**Table 2:** Comparison of patients’ characteristics between patients with EVT success and EVT failure

	EVT success (*n* = 8)	EVT failure (*n* = 2)	*p* value
Age, median	70 (56.25–76.25)	63 (62–64)	0.71
Female, *n* (%)	1 (12.5%)	0	0.99
BMI, median	23.5 (22–29.75)	27.75 (26–29.5)	0.51
ASA scores, *n* (%)			0.73
1	0	0	
2	2 (25%)	0	
3	3 (37.5%)	1 (50%)	
4	3 (37.5%)	1 (50%)	
Etiology of the perforation			0.28
Leak after duodenal suture	2 (25%)	2 (100%)	
Anastomotic leak	2 (25%)		
Iatrogenic perforation	2 (25%)		
After EMR	2 (25%)		
Prior treatment	Yes 4 (50%)	Yes 0	0.46
	No 4 (50%)	No 2 (100%)	
Latency between leak identification and EVT initiation, days, median (IQR)	7 (2–11)	2.5 (0–5)	0.22
Length of stay, days, median (IQR)	52 (21–83)	71 (25.5–103.3)	0.71
Size of the defect, centimeters, median (IQR)	2.5 (2–3.75)	2.25 (1–3.5)	0.71
Localization of the EVT, median (IQR)			0.37
Intraluminal	7 (87.5%)	1 (50%)	
Intracavitary	1 (12.5%)	1 (50%)	
Number of EVT changes, median (IQR)	3 (2–5)	6 (1–11)	0.88
EVT duration, median (IQR)	9 (7–30.75)	20.5 (5–36)	0.84
Additional treatment of leak (surgical or radiological drainage)	Yes 7 (87.5%) No 1 (12.5%)	Yes 2 (100%) No 0	0.99
Length of ICU stay, median (IQR)	21 (4.5–43)	17 (13–21)	0.90
In hospital complications (Clavien Dindo > 3a)	1 (12.5%)	2 (100%)	0.06
Mortality	1 (50%)	1 (50%)	0.37

*EMR* Endoscopic mucosal resection, *BMI* Body Mass Index, *ASA* American Society of Anesthesiologists, *ICU* intensive care unit

## Discussion

Management of duodenal perforations represents a clinical challenge with an associated mortality between 20 and 30% [21]. Surgical repair often increases the risk of another leak. Lang et al. showed that re-suturing of the leaks is associated with a mortality of 66.7% [22]. Endoscopic treatment with clips can directly close the defect, but this treatment option is often limited by the size of the perforation. EVT is a new endoscopic treatment option and can be used in two different ways—with the sponge placed either intraluminally or intracavitary [17]—and is limited neither by the size nor the localization of the defect (see Table 3).

**Table 3:** Outcomes of endoscopic vacuum therapy (EVT) for duodenal perforation described in the literature

Study	Year	Number of patients	Rate of closure	Number of changes	Number of EVT duration (days)
Loske et al. [1]	2019	11	100%	0–5	7–24
Glatz et al. [2]	2015	1	100%	3	20
Kelm et al. [3]	2017	1	100%	7	21
Hochberger et al. [4]	2016	1	100%	0	4

So far, only one case series and few case reports investigated the effectiveness of EVT in treating duodenal perforations: Loske et al. treated a cohort (*n* = 11) with duodenal leaks with EVT [23]. The authors described intraluminal EVT in nine patients, intracavitary in one and mixed-position EVT in one. In our series, eight patients had an intraluminal and two intracavitary treatments. In both studies, closure of the perforation could be achieved independently of the position of the sponge. This suggests that the exact localization of the sponge is less important as long as the position ensures a sufficient local vacuum effect. However, the recommendation for the optimal positioning has not yet been determined, and more studies are required to clarify the influence of the sponge position.

Successful leak closure was observed in eight of the ten patients (80%), whereas Loske et al. reported a success rate of 100%. They described different etiologies: eight suture leaks, one iatrogenic perforation associated with endoscopic retrograde cholangiopancreatography, one iatrogenic perforation after operative drain, and one anastomotic leak. In our series, the etiologies of the defects were leak after duodenal suture of perforated ulcer (*n* = 4), iatrogenic perforation after endoscopic resection (*n* = 2), iatrogenic perforation during surgery (*n* = 2), and anastomotic leak after upper gastrointestinal surgery (*n* = 2). These heterogeneous etiologies may explain the lower success rate: one treatment failure occurred in a cirrhotic, obese patient with multiple comorbidities. In his case, a retroperitoneal collection, which could not be drained by EVT, may have fueled an inflammatory process impairing the duodenal defect closure.

EVT alone may not be sufficient to treat complex and large duodenal defects. Surgical or radiologic drainage of any deep abdominal collections should be introduced additionally in the management of such patients. The other treatment failure involved a patient who developed septic shock with multiple organ failure, leading to death. One reason for unsuccessful EVT is that it is a slow-building therapy that requires hours to control the infection source. Therefore, it should not be applied in unstable septic patients, and exit strategies such as salvage surgery should always be considered.

When EVT is used in the esophagus, the most feared complication is bleeding, especially when the sponge is placed in the extraluminal position. Erosion of major vessels caused by the negative pressure therapy is the main concern [24]. We observed only two complications in our series: one case of bleeding that ceased with the stop of the negative pressure and one case of pneumothorax, which required surgical drainage. EVT in the duodenal region seems to be safe and associated with low morbidity. However, a larger patient cohort is needed to verify our results concerning the safety of this treatment.

Another important aspect is the timing of the treatment. In eight patients, EVT was initiated within the first 24 h after discovering the leak. In the other two cases, more than 20 days had passed before the initiation of EVT. In one case, this did not impede the closure, as it took only one sponge change and a treatment duration of 7 days to complete. In the other case, EVT failed. In the literature, one case of late EVT initiation for a leak from a gastroduodenal anastomosis was reported [25]. The leak was identified at postoperative day two. Direct repair was attempted twice, but the re-laparotomies were unsuccessful. Finally, 10 days after the discovery of the leak, EVT was placed using the pull-through technique. The defect and the fistula channel closed after six sponge changes over 21 days [25]. This case and our experience with late EVT initiation suggest that EVT can be used successfully to close duodenal defects, even when initiated late. However, further studies are required to assess if the latency of EVT introduction is a negative factor for the success of the treatment.

The number of sponge changes in our study varied from 1 to 15. This large difference could be explained by the various etiologies of the duodenal perforations, their varying size, different times of discovery of the leaks, and diversity in patients’ characteristics. The number of changes is higher than reported by Loske et al. (0–5) [23] and others (0–7) [24, 25]. In our study, one patient received 15 changes before complete closure. This higher number of changes suggests that, in individual cases, even a long treatment duration can successfully close duodenal defects.

In our study, eight patients had an ASA ≥ 3. Those patients are often critically ill, especially if they also suffer from adverse events such as gastrointestinal leaks. An advantage of EVT is that the treatment can be carried out under sedation in most cases. Avoiding additional general anesthesia is desirable for all patients, but especially critically ill patients, obese patients, and patients with significant comorbidities benefit from lower levels of invasiveness. We performed EVT under sedation in seven of the ten patients (70%) without any adverse events.

Since EVT in the duodenum is a new and unestablished treatment option, it is difficult to define the exact number of sponge changes beyond which EVT is considered futile. Therefore, close multidisciplinary patient monitoring involving the surgeon and the gastroenterology team is essential and exit strategies such as salvage surgery need to be continuously considered.

The strength of this study is its inclusion of a standardized material in each case, lowering the bias of different technique and materials. Limitations of this study are its retrospective design and the relatively small size of patient cohort.

In conclusion, EVT for duodenal leaks is safe and feasible, even in critically ill patients. The use of EVT in the duodenum might still help to control the clinical condition of these patients. A further evaluation and identification of selection criteria for this method is strongly recommended.
